# Mammographic Density Distribution of Healthy Taiwanese Women and its Naturally Decreasing Trend with Age

**DOI:** 10.1038/s41598-018-32923-z

**Published:** 2018-10-08

**Authors:** Wei-Chung Shia, Hwa-Koon Wu, Yu-Len Huang, Li-Sheng Lin, Dar-Ren Chen

**Affiliations:** 1 Cancer Research Center, Department of Research, Changhua Christian Hospital, Changhua, Taiwan; 2 Department of Medical Imaging, Changhua Christian Hospital, Changhua, Taiwan; 3 Department of Computer Science, Tunghai University, Taichung, Taiwan; 4 Department of Breast Surgery, The Affiliated Hospital (Group) of Putian University, Putian, Fujian China; 5 Comprehensive Breast Cancer Center, Changhua Christian Hospital, Changhua, Taiwan

## Abstract

We analysed typical mammographic density (MD) distributions of healthy Taiwanese women to augment existing knowledge, clarify cancer risks, and focus public health efforts. From January 2011 to December 2015, 88,193 digital mammograms were obtained from 69,330 healthy Taiwanese women (average, 1.27 mammograms each). MD measurements included dense volume (DV) and volumetric density percentage (VPD) and were quantified by fully automated volumetric density estimation and Box-Cox normalization. Prediction of the declining MD trend was estimated using curve fitting and a rational model. Normalized DV and VPD Lowess curves demonstrated similar but non-identical distributions. In high-density grade participants, the VPD increased from 12.45% in the 35–39-year group to 13.29% in the 65–69-year group but only from 5.21% to 8.47% in low-density participants. Regarding the decreased cumulative VPD percentage, the mean MD declined from 12.79% to 19.31% in the 45–50-year group versus the 50–55-year group. The large MD decrease in the fifth decade in this present study was similar to previous observations of Western women. Obtaining an MD distribution model with age improves the understanding of breast density trends and age variations and provides a reference for future studies on associations between MD and cancer risk.

## Introduction

Several studies identified mammographic breast density (MD) as an independent risk factor for breast cancer^1,2^. It is natural and accepted that breast density decreases with increasing age because of postmenopausal glandular breast tissue alterations^3^. The roles of MD are also included in estimations of adjuvant hormone therapy effects after surgery such as tamoxifen^4^ and the aromatase inhibitors^5^. Regardless of associations among MD, breast cancer risk, and endocrine therapy responses, it is important to have a realistic density model to improve accuracy in these related studies.

Prior studies of breast density among diverse age groups were based mainly on Western women^6,7^; it is interesting to evaluate different breast density distribution patterns between Asian and Western women. Prior Asian population-based studies well reported the association between dense mammograms and age^8–10^; however, assessments of longitudinal changes in breast density have used the area-based method^11^ to measure the two-dimensional area of dense breast tissue on digitized mammography based principally on a semi-automated procedure^1,12^. This operator-oriented analysis procedure results in unavoidable bias in dense region selection and limits the number of mammograms that can be studied.

Recently, full-field digital mammography (FFDM) has advanced the development of automated software that measures volumetric breast density in three dimensions^13,14^ and volumetric density measurements from FFDM also provide precise estimates of longitudinal changes in breast density^15,16^. It provides an objective and transparent method for density measurements and decreases disagreements among radiologists; this method can also handle large numbers of mammograms and would be useful in providing important prognostic information clinically^17^. In prior MD distribution studies based on Asian populations, the sample sizes of the cohorts usually ranged from 1,000 to 2,000 and the subjects’ ages ranged from 40 to 65 years. This inspired us to perform an MD distribution analysis based on a large number of FFDM examinations from an Asian population dataset (over 80,000 FFDMs) with all cohorts were from a single country (Taiwan). This can provide high-resolution MD analysis for each age range. We aimed to examine relationships and variations between age and breast density in healthy Taiwanese women by utilizing volumetric density measurements from FFDM with the goals of improving age-specific and risk-specific breast cancer screening guidelines.

## Results

### Characteristics of the Study Population

After applying the exclusion criteria, there were 69,330 healthy Taiwanese women who underwent mammography screening who did not have an overlapping record in the breast cancer registry from 2011 to 2015. These women were enrolled and data from 88,193 FFDM examinations were analysed. There were 15,269 participants who contributed multiple FFDM examinations during the study period (12,530 participants contributed two FFDMs, 2,739 participants contributed three or more FFDMs, and women had 1.27 mammograms on average during the 5-year period).

Table 1 shows the distribution of the study population according to each density grade and for each age group. Each age group or each 5-year interval included at least 800 participants aged 30 to 70 years. Only 273 participants were younger than 30 years; 372 participants were older than 75 years. The population aged 35 years to 69 years included 97.1% of the cohort. Therefore, further analyses and discussion in this study will focus on this age range.

**Table 1:** Distribution of VDG/BI-RADS grades of healthy Taiwanese women by age group.

*Age* (*y*)	Proportion (%) of participants who underwent screening using VDG/BI-RADS density categories for each age group			
	Grade a	Grade b	Grade c	Grade d
<30	0.7% (2/272)	4.4% (12/272)	12.5% (34/272)	82.4% (224/272)
*30–39*	0.2% (7/3,378)	5.2% (177/3,378)	25.8% (873/3,378)	68.7% (2,321/3,378)
*40–49*	0.2% (43/25,447)	6.2% (1,581/25,447)	35.8% (9,119/25,447)	57.8% (14,704/25,447)
*50–59*	0.4% (132/36,162)	17.0% (6,158/36,162)	51.0% (18,449/36,162)	31.6% (11,423/36,162)
*60–69*	0.7% (156/21,636)	32.5% (7,023/21,636)	53.0% (11,467/21,636)	13.8 (2,990/21,636)
>70	0.7 (7/1,019)	34.9% (356/1,019)	54.5% (555/1,019)	9.9% (101/1,019)
*Total*	**0**.**4%** (**347/88**,**193**)	**17**.**5%** (**15**,**399/88**,**193**)	**46**.**1%** (**40**,**654/88**,**193**)	**36**.**1%** (**31**,**794/88**,**193**)

The number of FFDM examinations in each age group is shown according to the density grade and its proportion of the total number in the group. The percentage (%) represents the proportion of the number of participants in each VDG/BI-RADS density category for each age group. The number of participants with a specified density is the numerator and the total number of participants in the specific age group is the denominator. The mammographic density grade was estimated in VDG by Volpara^TM^.

### Distribution of Mammographic Density

In this dataset, the median age at the time of mammography was 53 years, the median DV was 44.65 (95% confidence interval [95% CI], 44.45 to 44.85) and the median VPD was 12.3 (95% CI, 12.2 to 12.4). In the group aged 30 to 49 years, the “d” density grade represented 59.06% of the participants. The density grade “c” represented 51.76% of the participants aged 50 to 69 years. The proportion of participants with a density of “a” or “b” only represented 17.9% of all participants. Table 1 demonstrates the details of the proportions of participants in each density group and in each age group.

**Figure 1:**
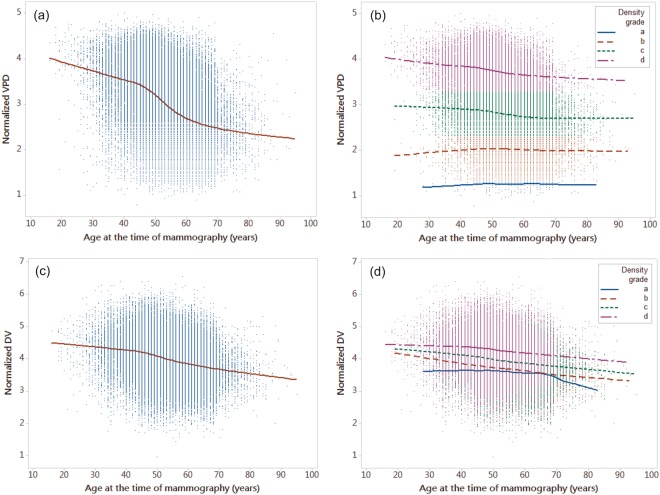
Normalized dense volume and percent volumetric density measures as functions of age at the time of mammography, showing a smoothed Lowess curve. The normalized dense volume in cm^3^ and volumetric density in percentage measurements are shown as a Lowess curve both ungrouped and grouped by density grade. (**a**) The Lowess curve of the mean normalized percent volumetric density measurements and ages of all participants. (**b**) The Lowess curve of the mean of the normalized percent volumetric density measurement and age grouped by density grade. (**c**) The Lowess curve of the mean normalized dense volume measurement and age for all participants. (**d**) The Lowess curve of the mean normalized dense volume measurement and age grouped by density grade. All breast density grades were estimated in VDG by Volpara^TM^. DV: volumetric density, VPD: percent volumetric density.

Figure 1a,c represent the Lowess curves for the normalized mean DV and VPD; these two curves have similar but not identical distributions. Table 2 shows the mean DV and VPD in each density group by age; the highest VPD also occurred in the age range from 35 to 39 years in the grade “c” and “d” groups and in the age range from 50 to 54 years in the grade “b” group. The variation in VPD from 35–39 years to 65–69 year in high density grade participants ranged from 12.45% (grade “c”) to 13.29% (grade “d”). For low density grade participants, this variation ranged only from 5.21% (grade “a”) to 8.47% (grade “b”). The highest DV occurred in the group aged 35 to 39 years regardless of the density group. This demonstrates that younger women tended to have the highest MDs.

**Table 2:** Mean volumetric density and percent volumetric density distributions by age group.

Age (y)	Mean of the DV and VPD with the 95% confidence interval			
	Grade a	Grade b	Grade c	Grade d
**35–39**				
DV	37.34 (9.82–64.86)	44.62 (41.22–48.03)	55.63 (53.34–57.92)	72.99 (71.00–74.98)
VPD	2.95 (2.57–3.33)	5.76 (5.58–5.95)	11.82 (11.65–12.00)	23.02 (22.78–23.25)
**40–44**				
DV	35.5 (26.60–44.40)	42.17 (40.38–43.96)	54.56 (53.47–55.65)	70.28 (69.15–71.40)
VPD	3.07 (2.79–3.35)	5.93 (5.82–6.04)	11.76 (11.66–11.85)	22.89 (22.75–23.04)
**45–49**				
DV	32.84 (28.62–37.06)	38.75 (37.89–39.61)	50.76 (50.20–51.33)	67.81 (67.11–68.51)
VPD	3.19 (3.11–3.28)	6.04 (5.99–6.10)	11.59 (11.53–11.64)	22.16 (22.07–22.25)
**50–54**				
DV	33.5 (30.97–36.05)	36.63 (36.09–37.17)	47.3 (46.84–47.76)	64.41 (63.62–65.20)
VPD	3.17 (3.11–3.23)	6.06 (6.02–6.09)	11.16 (11.11–11.20)	21.31 (21.21–21.41)
**55–59**				
DV	34.23 (31.48–36.97)	35.73 (35.28–36.19)	45.29 (44.85–45.73)	61.65 (60.55–62.75)
VPD	3.13 (3.07–3.19)	5.98 (5.95–6.02)	10.80 (10.75–10.84)	20.38 (20.26–20.51)
**60–64**				
DV	30.83 (28.74–32.93)	33.81 (33.41–34.21)	43.41 (42.93–43.88)	61.31 (59.77–62.85)
VPD	3.20 (3.16–3.25)	5.95 (5.92–5.98)	10.47 (10.42–10.52)	20.11 (19.94–20.29)
**65–69**				
DV	30.82 (28.55–33.09)	31.55 (31.13–31.98)	41.15 (40.56–41.74)	57.46 (55.18–59.74)
VPD	3.20 (3.15–3.24)	5.86 (5.82–5.89)	10.35 (10.29–10.42)	19.96 (19.71–20.22)

The mean volumetric density (in cm^3^) and percent volumetric density (%) with 95% confidence intervals of healthy Taiwanese women are shown for the defined age groups. Excluding ages under 35 years and over 70 years, each group represents a 5-year span. The mammographic density grade was estimated in VDG by Volpara^TM^.

DV: volumetric density, VPD: percent volumetric density.

### The Naturally Decreasing Trend of Mammographic Density

**Figure 2:**
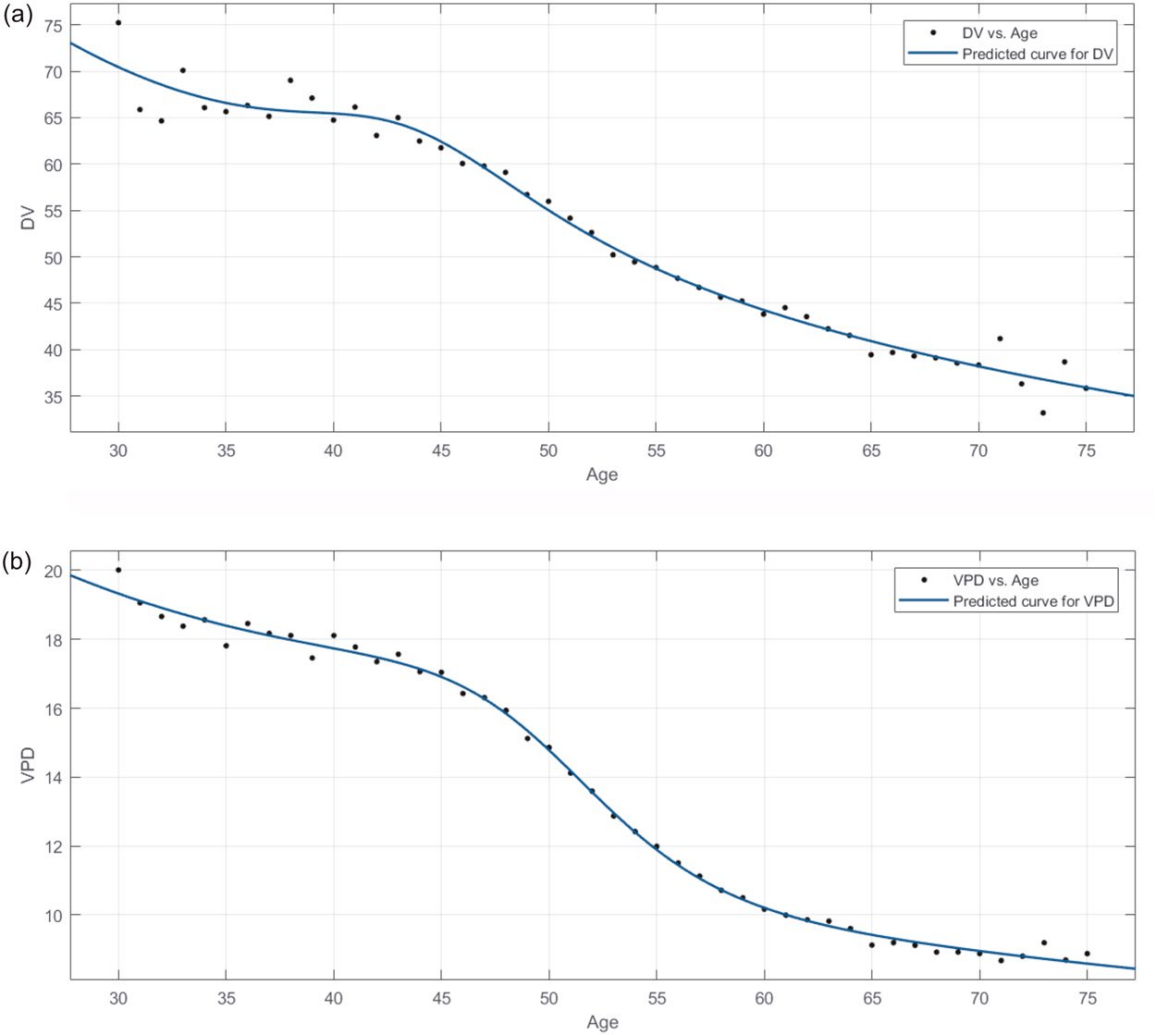
Distributions of mammographic volumetric density and percent volumetric density shown as fitted model curves. The mean volumetric density and percent volumetric density for each age were fitted in the rational model; the curve represents associations with age. (**a**) The fitted curve of mean volumetric density for each age. (**b**) The fitted curve of mean percent volumetric density for each age. Each data point represents the mean value for each age. DV: volumetric density, VPD: percent volumetric density.

Regarding the naturally decreasing trend in MD, the Lowess curves of the mean normalized VPD and DV (Fig. 1a,c) show that women had the highest MD before 45 years and had the steepest decline (slope of the curve) from age 45 to 55 years. After the age of 55 years, the declining DV and VPD trends were stable and slow and remained at a lower level. Regarding comparisons of VPD decline in each density group, Fig. 1b shows that high density grade participants (grades “d” and “c”, VPD >7.6%) had similar trends while low-density grade participants (grade “a” and “b”, VPD <7.5%) did not. Grades “b”, “c,” and “d” all demonstrated approximately similar declining trends in DV (Fig. 1d) and the DV declines in most women were similar. It cannot be ruled out that the reason that density grade “a” had a different trend than the others is the result of the lower number of participants (n = 347). Figure 2 shows the fitted curves for the mean DV and VPD in each age group to show the predicted curve and demonstrate the trend.

Table 3 represents the cumulative rate of decrease in VPD (as a percentage) with the age range for each density grade (the VPD in the group aged 35 to 39 years is the baseline) and shows the trend of declining MD in healthy Taiwanese women. In density grade groups “a” and “b”, the percentage of the cumulative rate of decrease in VPD was negative (this means that VPD increased with age) and presents the opposite trend as those observed in grades “c” and “d”. Table 3 also shows that the MD at age 50 years is a significant boundary and that the greatest decrease occurs from 45 to 55 years. In the 45–50-year and 50–55-year age groups, the proportion of decline in total mean MD was from 12.79% to 19.31%. This significant trend in the DV/VPD decline is also observed in the 45–55-year age range in Fig. 2.

**Table 3:** Naturally decreasing trend in mean percent volumetric density by age group and density grade.

Age (y)	Mean VPD (%)				Cumulative Decrease in VPD (%)			
VDG	a	b	c	d	a	b	c	d
35–39	2.95	5.76	11.82	23.02	—	—	—	—
40–44	3.07	5.93	11.76	22.89	−4.07	−2.95	0.51	0.56
45–49	3.19	6.04	11.59	22.16	−8.14	−4.86	1.95	3.74
50–54	3.17	6.06	11.16	21.31	−7.46	−5.21	5.58	7.43
55–59	3.13	5.98	10.80	20.38	−6.10	−3.82	8.63	11.47
60–64	3.20	5.95	10.47	20.11	−8.47	−3.3	11.42	12.64
65–69	3.20	5.86	10.35	19.96	−8.47	−1.74	12.44	13.29

This table focuses on the decreasing trend in the mean percent volumetric density in healthy women by age for each mammographic density grade from 35 to 69 years; each age group represents a 5-year interval. The mammographic density grade was estimated in VDG by Volpara^TM^.

VPD: percent volumetric density.

## Discussion

Regarding the naturally decreasing MD in healthy Taiwanese women, the DV/VPD Lowess curve in Fig. 1 is observed to be non-linear. Figure 2 shows the fitted curve for DV/VPD; the fitted model curves had approximately the same trends when compared with the Lowess curves visually. This will affect many assumptions such as the assumption that the MD decline with age is linear. The most severe trend in declining density (the steepest slope of the predictive curve between any two age ranges in Fig. 2) was from 45–50 years to 50–55 years. This indicates that postmenopausal changes are indeed the dominating factor in MD decline. Although there was a lack of detailed information or a questionnaire to confirm the actual date of or age at menopause in this study, the average age at menopause among Taiwanese women reported in a previous study was usually in the fifth decade of life and between 45 and 55 years of age for most women^18,19^; this is a reasonable assumption and was used as a reference in this study.

The large decrease in MD in the fifth decade of life in the present study was similar to previous observations in Western women^6,11^. By comparing the Lowess curve with the fitted model curve, the association between MD and age in the present study was also similar to that of a meta-analysis combining cross-sectional MD data from 22 countries worldwide^12^ and a recent Australian population-based study^1^. This means that the previous conclusion also benefits Taiwanese women by having a similar distribution and decreasing MD trend with increasing age. However, Table 3 shows that the VPD in low density grade participants increased with age, and the top level of the VPD has the opposite trend to that of grades “c” and “d”. The Lowess curve for the VPD according to each density grade and age range also presents this trend in Fig. 1. It shows that participants who have low density grades may have different MD distributions with age than others and indicates that it includes different assumptions regarding cancer risk.

In the present study, the typical MD distribution in healthy Taiwanese women was shown, as were the relationship and declining trend between MD and age. The results of this study improve the understanding of MD trends and variations with age in Taiwanese women and provide a reference for future related studies on associations between MD and cancer risk. Important limitations to the present study include a lack of information such as the BMIs and menopause statuses of the participants; therefore, the effects of menopause and BMI on MD were not estimated or modelled. This is future work that may result in more information to distinguish different MD distributions in the Taiwanese population than those observed in the present study. We hope that this study will provide sufficient information about normal MD distributions in Taiwanese women.

## Methods

### Study

This study was based only on raw FFDM data enrolled from an outpatient clinic and mammography screenings during the period from January 2011 to December 2015; 102,094 FFDM examinations from 77,427 women stored at the institute were subsequently analysed. The ages of the participants ranged from 16 to 95 years. All participants underwent the same procedure and all FFDM examinations were performed at the same institute. No further questionnaire or other data were added to assist in obtaining information such as the body mass index (BMI), menopausal status, or family history.

**Figure 3:**
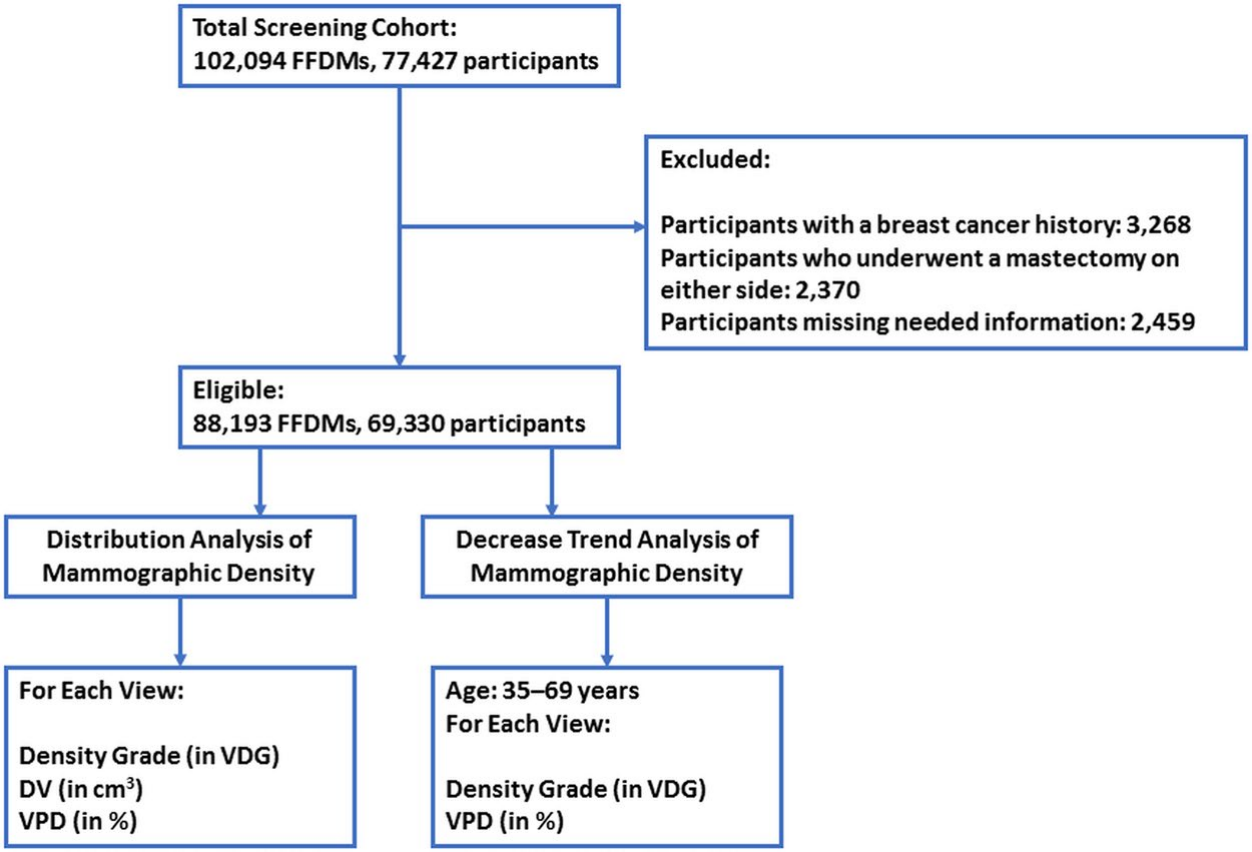
Flow chart showing FFDM data exclusion and enrolment.

The exclusion criteria were participants with breast cancer, those who underwent a mastectomy on either side, FFDM examinations that lacked the information required for the analysis (e.g., age, study date); and non-four-view mammograms. Participants were permitted to contribute multiple mammograms because this dataset also includes national mammography screening and follow-up data (free breast screening every 2 years for healthy women aged over 40 years in Taiwan). Each mammogram was compared with the records of the breast cancer registry at the institute to exclude participants with a breast cancer history and to ensure that participants met the inclusion criteria (n = 3,268, recorded from January 2011 to December 2015). Detailed exclusion criteria and the workflow for FFDM data pre-processing are shown in Fig. 3.

This cross-sectional retrospective study was approved by the Institutional Review Board of Changhua Christian Hospital, Changhua, Taiwan (No. 160814 and No. 171217). The requirement for informed consent was waived by the ethics committee because of the study’s retrospective nature. All experiments were performed in accordance with relevant guidelines and regulations.

### Mammographic density measurements

All mammograms were obtained from the Department of Medical Imaging at Changhua Christian Hospital, Changhua, Taiwan. All standard two-dimensional digital mammograms used in this study were obtained using FFDM systems including Senographe Essential/Senographe DS (GE Medical Systems, Milwaukee, WI, USA) (79.3% of all mammograms), Mammomat Inspiration (Siemens AG Healthcare, Erlangen, Germany) (14.1% of all mammograms), and Selenia Dimensions (Hologic, Inc., Bedford, MA, USA) (6.6% of all mammograms). Each FFDM examination was confirmed to be a bilateral four-view examination before analysis.

The automated measurement of MD and MD grading were performed using Volpara^TM^ software version 1.5.1 (Volpara Health Technologies, Wellington, New Zealand). Briefly, through a specific algorithm that models X-ray physics, the total breast volume (in cm^3^), the overall dense breast volume (DV) (in cm^3^) and volumetric density percentage (VPD) estimates were produced^20^. The VPD was obtained from the DV divided by the total breast volume and multiplied by 100. The DV and VPD in the present study were calculated by averaging the left and right sides.

MD grades were reported using the Volpara Density Grade (VDG). The VDG classification is based on quantitative volumetric measurements of fibroglandular and fatty tissue to calculate the VPD (a: less than 4.5%; b: 4.5% to 7.5%; c: 7.6% to 15.5%; d: higher than 15.5%) and has different thresholds from the Breast Imaging-Reporting and Data System (BI-RADS) fifth edition^21^. The VDG has already been proven to have a good correlation with radiologist-assigned BI-RADS density categories^22^; therefore, we did not measure the variance between the VDG and the agreement of the radiologists or combined radiologist-assigned BI-RADS score data.

### Statistical Analysis and Curve-Fitting Analysis

The MD measurements were transformed to meet the assumption of a mixed-effects model in which the residuals have an approximately normal distribution. The Box-Cox transform method^23^ was used to identify and transform the MD measurements by finding an optimal lambda value (λ = 0.025 for DV, λ = 0.133 for VPD) so that their residuals were approximately normally distributed. Locally weighted scatterplot smoothing (Lowess) curves were used to fit the normalized DV and VPD to age (bandwidth = 0.5). It was plotted on the basis of the smoothed VD/VPD and age by applying the greatest weight to the point of interest and decreasing weights for points further away based on their x-axis distance to the point of interest in the dataset^24^. The independent t test was used to test for differences between each age group and p-values less than 0.01 were considered statistically significant.

Curve-fitting and regression model analyses were used for an investigation of the association between DV/VPD and age. The population aged from 30 years to 75 years (which included 98.2% of the cohort) was added to the curve-fitting analysis to obtain a more suitable curve to present the MD distribution of younger women (younger than 40 years). The curve-fitting performance is evaluated according to the model with the minimum residual and highest R^2^ value. Rational models with a numerator of degree two and denominator of degree four (rat24) had the best fits after comparisons with several regression models such as linear and cubic/polynomial models and thus were chosen to describe the curve (R^2^ = 0.9782/root mean squared error [RMSE] = 1.7424 for DV fitting; R^2^ = 0.9964/RMSE = 0.2357 for VPD fitting). The formula for the rational models is described in equation (1) as follows:

1 $${\rm{f}}({\rm{x}})=\frac{p1{x}^{2}+p2x+p3}{{x}^{4}+q1{x}^{3}+q2{x}^{2}+q3x+q4}$$

where x represents age. When predicting the mean VPD, model-related parameters were described as follows: *p*1 = 4.209e^6^, *p*2 = 3.574e^5^, *p*3 = 2.56e^6^, *q*1 = 5.805e^4^, *q*2 = 3.687e^5^, *q*3 = 1.445e^5^ and *q*4 = 1.931e^5^. When predicting the mean DV, model-related parameters were described as follows: *p*1 = 5.68e^6^, *p*2 = 7.957e^6^, *p*3 = 5.567e^6^, *q*1 = 2.328e^4^, *q*2 = 1.55e^5^, *q*3 = 1.89e^5^ and *p*4 = 1.079e^5^. Fitted model curves were also compared with Lowess curves visually.

All statistical analyses and regression model analyses were performed using Minitab 18 statistical software (2018) (Minitab, Inc., State College, PA, USA). The curve-fitting for rational models was performed using MATLAB and the Statistics Toolbox Release 2017b (The MathWorks, Inc., Natick, MA, USA).

## Data Availability

The datasets generated during and analysed during the current study are not publicly available due to restrictions of IRB and institute but are available from the corresponding author on reasonable request.
